# Trabecular bone texture analysis of conventional radiographs in the assessment of knee osteoarthritis: review and viewpoint

**DOI:** 10.1186/s13075-021-02594-9

**Published:** 2021-08-06

**Authors:** Ahmad Almhdie-Imjabbar, Pawel Podsiadlo, Richard Ljuhar, Rachid Jennane, Khac-Lan Nguyen, Hechmi Toumi, Simo Saarakkala, Eric Lespessailles

**Affiliations:** 1 EA 4708- I3MTO Laboratory, University of Orleans, Orleans, France; 2 Translational Medicine Research Platform, PRIMMO, Regional Hospital of Orleans, Orleans, France; 3 Tribology Laboratory, School of Civil and Mechanical Engineering, Curtin University, Bentley, WA 6102 Australia; 4 ImageBiopsy Lab, Vienna, Austria; 5 Department of Rheumatology, Regional Hospital of Orleans, Orleans, France; 6 Physics and Technology, Research Unit of Medical Imaging, University of Oulu, Oulu, Finland; 7 Department of Diagnostic Radiology, Oulu University Hospital, Oulu, Finland

**Keywords:** Trabecular bone texture, Knee osteoarthritis, Conventional radiography

## Abstract

**Background:**

Trabecular bone texture analysis (TBTA) has been identified as an imaging biomarker that provides information on trabecular bone changes due to knee osteoarthritis (KOA). Consequently, it is important to conduct a comprehensive review that would permit a better understanding of this unfamiliar image analysis technique in the area of KOA research.

We examined how TBTA, conducted on knee radiographs, is associated to (i) KOA incidence and progression, (ii) total knee arthroplasty, and (iii) KOA treatment responses. The primary aims of this study are twofold: to provide (i) a narrative review of the studies conducted on radiographic KOA using TBTA, and (ii) a viewpoint on future research priorities.

**Method:**

Literature searches were performed in the PubMed electronic database. Studies published between June 1991 and March 2020 and related to traditional and fractal image analysis of trabecular bone texture (TBT) on knee radiographs were identified.

**Results:**

The search resulted in 219 papers. After title and abstract scanning, 39 studies were found eligible and then classified in accordance to six criteria: cross-sectional evaluation of osteoarthritis and non-osteoarthritis knees, understanding of bone microarchitecture, prediction of KOA progression, KOA incidence, and total knee arthroplasty and association with treatment response. Numerous studies have reported the relevance of TBTA as a potential bioimaging marker in the prediction of KOA incidence and progression. However, only a few studies have focused on the association of TBTA with both OA treatment responses and the prediction of knee joint replacement.

**Conclusion:**

Clear evidence of biological plausibility for TBTA in KOA is already established. The review confirms the consistent association between TBT and important KOA endpoints such as KOA radiographic incidence and progression. TBTA could provide markers for enrichment of clinical trials enhancing the screening of KOA progressors. Major advances were made towards a fully automated assessment of KOA.

## Introduction

Osteoarthritis (OA) is the most common type of arthritis and is among the leading cause of impaired mobility and chronic pain, affecting almost half of the population aged 65 years or older worldwide [1].OA is considered as the most prevalent disorder of articulating joints in humans [2]. Knee, hip, and hand OA are the most common forms of the disease [3], with knee being the primary joint of interest [4, 5]. Reducing pain and decreasing the progression of joint damage in patients with knee OA (KOA) is still a challenging task [6]. Early detection and assessment of KOA prognostic factors are crucial for developing management and treatments that aim at preventing irreversible damage to the knee joint leading to arthroplasty. Therefore, one of the primary goals of imaging biomarkers is to identify patients at high risk of KOA progression [7].

Trabecular bone texture analysis (TBTA) involves the examination of the vertical and horizontal trabeculae of a predefined bone region of interest (ROI). Previous studies have reported that TBTA is able to predict not only the incidence of radiographic OA [8–10] but also the radiographic progression of the disease [8, 11–15].

TBTA has been identified as an imaging biomarker that provides information on trabecular bone changes due to KOA. Consequently, it is important to conduct a comprehensive review that would permit a better understanding of this unfamiliar image analysis technique in the area of KOA research. The aims of this article are twofold: to provide (i) a review of the studies conducted on radiographic KOA using TBTA, and (ii) a viewpoint on future research priorities.

## Methods

This review highlights original research articles published between June 1991 (publication date of the seminal paper by [16]) and March 2020, on traditional and fractal-based texture analysis of OA-related changes in trabecular bone. The SANRA criteria were used as a framework of quality assurance of the present manuscript [17]. We narrowed our focus down as follows:

- Site investigated: knee
- Imaging modality: conventional radiography
- Image processing method: texture analysis
- Target subject: human

### Search strategy

**Fig. 1:**
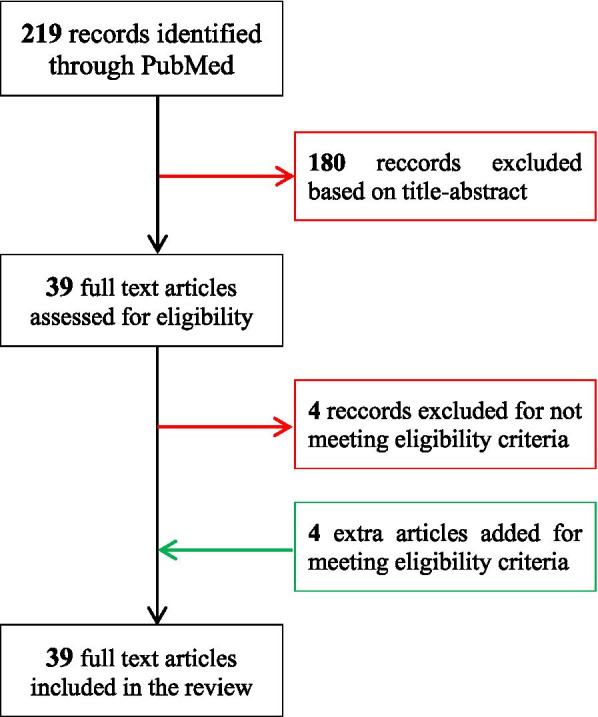
Flow diagram for the article selection

Our search strategy is illustrated in Fig. 1. Literature searches were performed in the PubMed electronic database via MeSH with the publication date set between June 1st, 1991, and March 31st, 2020. The search keywords and Medical Subject Headings used were:

(“Osteoarthritis”[Mesh] OR Osteoarthriti*) AND (“Osteoarthritis, Knee”[Mesh] OR “Knee”[Mesh] OR “Knee Joint”[Mesh] OR “Knee Prosthesis”[Mesh] OR Knee) AND (“Radiography”[Mesh] OR “X-Rays”[Mesh] OR Radiograph* OR Xray* OR X-Ray*) AND (“Cancellous Bone”[Mesh] OR Trabecula* OR TBT OR Cancellous Bone) AND ((“1991/06/01”[PDat]: “2020/03/31”[PDat]) AND (English[lang])).

The reference lists of all the included studies were screened to identify any relevant missing studies and discard the wrongly selected ones. The search was limited to humans and English language full-text.

The PubMed database search resulted in 219 papers. After scanning the titles and abstracts in Zotero, 39 papers were used for further investigation.

### Eligibility criteria and selection process

Articles were considered eligible if they presented quantitative work on TBTA of KOA using conventional radiography. Exclusion was not applied to statistical methods. Exclusion criteria included studies related to other than human KOA, imaging techniques other than conventional radiography, and studies without TBTA. Non-peer-reviewed articles, dissertations, abstracts, conference proceedings, commentaries, and letters to the editor were also excluded.

Two authors (A.A., E.L.) performed the selection process independently. Agreement was achieved by discussion. The full texts of potential articles were then retrieved and screened using the same procedure.

## Results

### Study selection

The search strategy identified 219 studies. Based on title or abstract screening, 180 studies were excluded since they did not meet the eligibility criteria. After full-text screening, an additional 4 studies were excluded. The subsequent full-text assessment resulted in 35 records that were found eligible for the comprehensive review. An additional 4 records were identified by checking the references and were included in the current review.

### Data extraction

Two authors (A.A., E.L.) independently classified the selected studies. Any disagreements between the reviewers were resolved by a consensus meeting with the third author (K.N.). The five criteria used in the classification process were (see Table 1 for the list of studies included in each category):

1. Cross-sectional quantitative evaluation between OA and non-OA knees
2. Understanding the microarchitecture of subchondral bone
3. Prediction of OA progression
4. Prediction of radiographic OA incidence
5. The association of TBTA with other endpoints

**Table 1:** List of studies included in the 6 categories

*N*°	**Category**	**Reference listing**
1	Cross-sectional quantitative evaluation between OA and non-OA knees (*24 articles*)	[16, 18–40]
2	Understanding the microarchitecture of subchondral bone (*2 articles*)	[41, 42]
3	Prediction of OA progression (*8 articles*)	[8, 11–15, 43, 44]
4	Prediction of radiographic OA incidence (*3 articles*)	[8–10]
5	The association of TBTA with other endpoints (*2 articles*)	[45, 46]

### What is known about TBTA and KOA

#### Cross-sectional evaluation of changes in trabecular bone texture (TBT) between OA and non-OA knees

Several methods have been proposed for the analysis of TBT aimed at discriminating knees with and without radiographic OA. In a seminal paper published in June 1991, Lynch et al. [16]introduced a new method based on fractal dimension (FD) and applied it to digitized images of knee macroradiographs. They demonstrated that subchondral tibial TBT on these images was fractal above a certain resolution [16]. They also demonstrated that the fractal signature (FS), a numerical measure of image texture that takes into account the influence of image resolution on the FD, was able to quantify OA changes in subchondral tibial bone texture roughness and directionality [25]. After further exploring the quantitative changes in subchondral trabecular bone associated with KOA in the medial compartment [19, 20], a larger study enrolling 110 patients with KOA compared to 27 non-OA volunteers was conducted [26]. In this study, it was reported that the trabecular bone structure quantified using FS analysis (FSA [25]) was significantly different both in the medial and lateral compartments compared to the non-OA control group [26]. The key finding was an increased FS of vertical trabeculae in OA, consistent with an “osteoporosis like” change at the subchondral ROI [26]. The development of FSA was carried out on high resolution macroradiographs. A further study was therefore conducted to show that the analysis is able to produce relevant discriminant results between OA and non-OA patients on conventional low-resolution radiographs [29]. It was confirmed, though in a small sample of OA (*n* = 24) and non-OA (*n* = 10) patients, that FSA could also detect significant disease-related changes in subchondral bone microarchitecture using the standard lower resolution radiographs [29]. The potential link between osteophytes and juxta-articular bone was explored by means of FSA in a cross-sectional study of 60 patients with KOA and 21 healthy non-OA volunteers [28]. The findings that emerged from this study suggest that the architectural organization of trabecular bone in the marginal regions is related to the size of the osteophytes [28].

The performance of a modified Hurst orientation transform (HOT) method, originally proposed by Podsiadlo et al. [47], was evaluated under different imaging conditions [32]. It was found that the method was sensitive to image noise, blur, and magnification, but relatively unaffected by projection angle and exposure. Three other methods (fractal signature Hurst orientation transform (FSHOT), variance orientation transform (VOT), and blanket with rotating grid (BRG)), were developed to calculate FS in all possible dimensions [40]. The objective was to overcome the limitation of the FSA and HOT methods that are able to calculate FS only in the vertical and horizontal directions. The three FS methods were evaluated using computer-generated isotropic fractal surface images with known FDs and X-ray images of a human tibial head. Results showed that, overall, the VOT method was more accurate in measuring bone texture roughness and anisotropy, and also less sensitive to imaging conditions and translation of bone region than the other two methods. The VOT method was also evaluated in the determination of differences in TBT between OA and non-OA knees [31, 39]. The study by Wolski et al. conducted on a small-size population of 46 individuals (26 controls (without OA) and 26 cases (Kellgren & Lawrence (KL) grade ≥ 2) matched by sex, age, body mass index (BMI), and compartment) showed that the FD of cases was lower than that of the controls. The study also showed that, unlike HOT, the VOT method detected changes in texture roughness over a wide range of trabecular sizes and along the roughest part of the tibial bone and texture anisotropy at individual trabecular sizes. Furthermore, the VOT method was used to detect differences in TBT between patients without KOA (KL grade less than 2) but with and without cartilage defects on a different and slightly larger cohort of 48 individuals [38]. Cartilage defects were graded on MRI images using a 5-point scale in relation to the thickness of the cartilage and the level of exposure of subchondral bone. At trabecular sizes of 0.3–0.4 mm, the results showed that in both medial and lateral compartments values of FS calculated in the vertical (FS_V_) and horizontal (FS_H_) directions were higher for cases as compared to controls. An alternative method was proposed based on distances between images quantified by a signature dissimilarity measure (SDM) [36]. The method was found to be invariant to image exposure, magnification, noise, and image size (> 64 × 64 pixels). However, it was sensitive to small image sizes, projection angle, and anisotropy. The performance of the SDM method was evaluated in the detection of OA between 17 healthy and 37 OA subjects. A classification accuracy of 78.8% was achieved by the SDM method.

While the research group of Podsiadlo et al. [32, 37–40] focused mainly on fractal-based techniques, the research group of Saarakkala et al. [21–24] proposed non-fractal-based techniques. The ability of radiography-based bone texture analysis to separate patients with different KL grades (1–4) with and without articular cartilage damage from those with and without bone marrow lesions was evaluated in the medial subchondral bone tibia [23]. Although the subjects were symptomatic and controls could have OA-related changes, TBTA was able to discriminate the cartilage damage from BML. Non-fractal-based techniques for texture analysis have also been used to discriminate patients with different KL grading scores. In [24], predefined parameters obtained from both Laplacian-based and Local Binary Pattern (LBP)-based images were studied and shown to be able to separate subjects in two groups according to their KL scores. The LBP method performed better.

Further studies have demonstrated that TBT parameters, measured in plain radiographs from KOA patients, are related to cartilage composition assessed by MRI. In [22], a weak relationship between TBT parameters selected in the medial subchondral bone and T2 relaxation time values of the medial tibial cartilage was found. However, no difference was observed between tibial cartilage composition and bone structural parameters in the medial tibial bone. In a sample of 109 subjects, Hirvasniemi et al. evaluated the importance of post-processing algorithms in the assessment of bone density and texture on plain radiographs [21]. The study confirmed the mandatory use of calibrated grayscale values to measure bone density. It was reported that machine learning plays an important role in exploring clinical covariates and bone density and texture, with the aim of building models that discriminate efficiently between patients with or without KOA [21].

Several methods have been proposed to demonstrate the sensitivity of TBT changes induced by treatments or other factors modifying the progression of KOA. Roemer et al. [33] studied the behavior of the trabecular bone structure extracted from radiographs of subjects exposed to increased joint point loading such as that experienced by young active athletes. Associations of FSs calculated on bone texture in the horizontal and vertical directions with four risk factors, namely athletes, gender, ACL surgery, and age, were examined. The study included 685 patients of whom 19.7% were athletes, 81.2% were male, and 8.8% had previous ACL. Mean age was 28.5 years (SD ± 6.5). Although significant differences in FSs in both compartments and in both the vertical and horizontal directions were observed, the study included a heterogeneous population, as acknowledged by the authors. Anterior cruciate ligament (ACL) injury may contribute to the development of a post-traumatic OA (PTOA) [48]. In a study of a small sample of 19 patients with ACL rupture, the radiographic changes in association with joint space width (JSW) were examined. Macro-radiographs of the patients’ knees (the diseased one and the uninjured one) were acquired at a mean time from injury of 34.3 (95% confidence interval (26.9 to 41.7) months [18]. Although no statistical difference in JSW or in subchondral cortical plate thickness was observed, there was a significant decrease in FD for ACL ruptured knees in the horizontal trabeculae of the medial compartment compared to the uninjured ones. To the best of our knowledge, the ability of these microarchitectural changes, detected about 4 years after injury, to predict the ongoing incidence of PTOA has not yet been evaluated. The trabecular orientation in the distal and proximal tibia was investigated by Sampath et al. [34], considering 90 OA patients, mostly with varus alignment. Significant correlations between the mechanical axis (leg alignment), calculated as the angle between the femoral and tibial axes, and trabecular orientation, determined using structure tensor-based analysis, in both distal and proximal tibia, were observed. In this study, interestingly, the trabecular anisotropy was observed to be greater in the medial tibiofemoral compartment than in the lateral side, consistent with the type of studied cases, where tibiofemoral KOA is generally more commonly observed in the medial than in the lateral compartment. In a study including 9 patients with symptomatic KOA, Miller et al. [30] investigated the differences in TBT between knees with and without a treatment based on a minimally invasive joint unloading implant. Significant changes in vertically oriented trabeculae, as reflected by FSA, were observed in this 2-year study. Results demonstrated the relationship between joint loading and subchondral trabecular structure and orientation, particularly in the medial subchondral compartment. In a recent study, the VOT method was used to evaluate changes in TBT after arthroscopic partial meniscectomy (APM) in non-radiographic OA knees (KL scores less than 2 in either knee) [37]. This study included 21 subjects (aged 35–55 years). Radiographs were acquired before APM and one year after surgery. The primary result was an increase in the ratio of medial-to-lateral TBT roughness in the knees undergoing APM as compared to the non-injured knees, before and after APM. These results might be useful in the prediction of KOA. A previous study evidenced that arthroscopic APM is an important factor of KOA risk for patients over 40 years old [49].

Periarticular and subchondral bone mineral density (BMD) may reflect a complex cross-talk between articular cartilage and subchondral bone [50] and could predict KOA progression [51]. In a pilot study of 11 KOA and 11 non-KOA tibiae, differences in trabecular bone organization and BMD between site-matched ROIs were analyzed [27]. No differences in BMD were found. Trabecular organization measured by FSA was significantly different in OA as compared to non-OA tibiae. This indicated that TBT was more sensitive than BMD to OA changes in bone features [27]. Periprosthetic dual-energy X-ray absorptiometry has been proposed to assess bone structural changes occurring after total knee arthroplasty (TKA) [52]. Moreover, FSA of macroradiographs of knees with total condylar knee prosthesis fixed with cement might quantify the longitudinal trabecular bone changes related to total knee arthroplasty. The trabecular changes were much greater in the lateral than in the medial compartment [53].

#### 2D-3D correlation of TBT parameters

The texture analysis of bone microarchitecture is based on plain knee radiographs, which are 2D projections of a 3D microstructure of trabecular bone. Consequently, the third dimension is lost and this may create ambiguities in analyzing X-ray images in spatial terms. Nevertheless, it has been reported that the textural bone parameters were associated significantly with 3D trabecular bone parameters at various bone sites including femur [42] and calcaneus [54, 55]. These studies, however, focused mainly on osteoporosis-related changes and not on OA-induced changes. Thus, Hirvasniemi et al. investigated relationships between 2 and 3D bone structure parameters calculated for the site-matched ROIs of subchondral bone selected on plain radiographs and 3D μCTs of the proximal tibia respectively [41]. Despite a lack of any diagnosed joint disease of the cadaver knees and a lack of usual soft tissue surrounding the knee during the radiographic process, 2D bone structure parameters correlated significantly with the 3D μCT parameter. There were strong inverse correlations between the FD calculated for vertical structure and the trabecular spacing [41].

#### Prediction of radiographic OA progression

The ability of TBTA to predict the progression of KOA has been evaluated in several papers [8, 11–15, 43, 44]. The main studies are summarized in Table 2 and they show that FSA is the most popular method.

**Table 2:** Summary of the main studies related to the prediction of OA progression

	Cohort name (*m*, *n*)	Period	Definition	Progression	Major findings
Messent et al. 2005 [12]	King’s College London (RG, 40)	23	KL ≥ 2	ΔJSN_M_ ≥ 1	No link was found between the degree of subchondral trabecular bone changes, analyzed by fractal signature analysis (FSA), and the rate of cartilage destruction, quantified by the annual rate of joint space narrowing (JSN)
Kraus et al. 2009 [14]	POP study (CR, 123)	36	1 ≤ KL ≤ 3	ΔJSN_M_ ≥ 1	Baseline trabecular bone texture analysis (TBTA), measured by FSA, of the medial tibial plateau was predictive of medial knee JSN progression. The predictive model with maximum effectiveness combined FSA, knee alignment, traditional covariates, and bone mineral content at baseline (AUC 0.79)
Woloszynski et al. 2012 [43]	University of Western Australia (CR, 50)	48	KL ≥ 2	ΔJSN_M_ ≥ 1	Baseline TBTA, measured by the dissimilarity-based multiple classifier (DMC), of the tibial plateau was predictive of knee JSN progression (accuracy = 0.80, specificity = 0.82, and sensitivity = 0.78)
Woloszynski et al. 2012 [8]	Lund University (CR, 105)	48	KL ≥ 2	ΔJSN_M_ ≥ 1	Baseline TBTA, measured by the signature dissimilarity measure method (SDM), of the tibial plateau was predictive of medial knee JSN progression (AUC = 0.77)
Kraus et al. 2013 [13]	Pfizer study (CR, 58)	12–24	2 ≤ KL ≤ 3 JSW_M_ ≥ 2 mm	ΔJSW_M_ ≥ 5% ΔJSA_M_ ≥ 5%	Bone trabecular integrity (BTI), measured by FSA, of the medial tibial plateau at baseline was predictive of 5% change in OA progression of joint space area (JSA) (AUC = 0.85)
Janvier et al. 2017 [11]	OAI (CR, 1124)	48	2 ≤ KL ≤ 3	ΔJSN_M_ ≥ 1	Baseline TBTA of the tibial plateau at baseline was predictive of medial knee JSN progression. Best model included clinical covariates, JSN and TBTA, measured by Whittle estimator (Whe), (AUC = 0.75)
Kraus et al. 2018 [15]	FNIH (CR&RG, 579)	24–48	1 ≤ KL ≤ 3 JSN_L_ < 2	ΔJSW_min ≥ 0.7	Using baseline TBTA, medial minimum joint space width (JSW) progression was characterized by thinner vertical and thicker horizontal trabeculae (best C-statistic = 0.649)

*n* total number of included subjects, *m* image acquisition modality, *POP* prediction of osteoarthritis

The usual clinical covariates of interest used to select patients with progressive OA include age, gender, and BMI. JSW and semi-quantitative KL grading are also commonly used in clinical trials (risedronate [46, 56], SEKOIA [57]). Fractal and non-fractal-based texture analysis methods can play an important role in the selection of patients as they provide the numerical measures of OA-induced bone changes.

In a 2-year longitudinal study conducted in 40 patients, FSA was used to measure prospectively structural bone changes observed in the subchondral and subarticular regions of their diseased proximal tibia. The main finding was the decrease in the FD of both horizontal and vertical trabeculae in OA knees compared to the non-OA ones [12]. It was also found, however, that knees with slow or detectable joint space narrowing (JSN) progression could not be separated. This is probably due to the limited size of the patient sample.

Based on a group of 30 subjects with advanced KOA (17 men and 13 women) [44], using TBT parameters, a quantification of subchondral tibial sclerosis in the setting of KOA progression was proposed. It was found that a higher grade of JSN was associated with a lower porosity and a higher number of free trabecular ends. BMI, gender, age, and knee alignment angle had strong effects on this association. However, no apparent correlations were found between TBT parameters and knee alignment angle.

High reproducibility in quantifying bone sclerosis using Tb.Sp and its significant relationship with JSW demonstrated potential for assessing OA progression.

In a cohort of 138 participants, it was reported that the model based on baseline TBT parameters outperformed those that included only JSW and traditional clinical covariates [14]. In another cohort of 60 subjects, the predictive power of TBT parameters calculated by FSA was studied [13]. KOA progression was evaluated in this 24-month longitudinal study using high-quality digital radiographs obtained from a clinical trial. As determined by both MRI and plain radiography outcomes, baseline trabecular bone parameters were able to assist in the prediction of OA progression [13].

In a relatively large population including 194 KOA cases with both pain and radiographic progression and 406 comparators (knees without OA), the authors concluded that TBT parameters could be useful as an OA trial enrichment strategy targeting patients at high risk of disease progression [15].

The ability of TBT to predict KOA progression was also evaluated using the OAI dataset [11]. In this large population including 1124 patients, a few novelties were proposed. First, a patchwork of ROIs was proposed that covered both the medial and lateral subchondral bone regions. Second, TBTA was performed using three different methods: FSA, quadratic variations (Var) and Whittle (Whe). Third, a sensitivity analysis was conducted to evaluate the potential effect of center (X-ray devices) on the predictive capacity of TBT. Their predictive model included the clinical covariates (age, gender, BMI), JSN, and TBT parameters with an area under the ROC curve (AUC) of 0.77. The AUC is usually used to indicate the overall accuracy of a test according to its sensitivity and its specificity (higher accuracy as the value approaches 1).

In another study, Woloszynski et al. [43] proposed a dissimilarity-based multiple classifier (DMC) system that uses distances between X-ray images and a diverse classifier ensemble. This system enables prediction of KOA without the calculation and selection of TBT features. Based on a sample of 50 subjects, the system predicted KOA progression defined as an increase in the sum of JSN and osteophytes over 4 years with an accuracy of 80% [43].

For OA progression, all the studies reviewed included patients with KL = 2 and KL = 3 at baseline. Some of them also included patients with KL = 1 or KL = 4 at baseline. In terms of the TBTA method, FSA was the most widely used method.

#### Prediction of radiographic OA incidence

The task of automatically assessing KOA severity can be approached as an image classification problem [35]. For example, an algorithm, called Weighted Neighbour Distance using a Compound Hierarchy of algorithms Representing Morphology (WND-CHRM) [35], was designed to identify biometrics that are capable of predicting KOA in knees without radiographic OA at baseline. The WND-CHRM generates a set of 34 descriptors from Tamura [58] and Haralick [59] texture features. In 123 pairs of X-ray knee images, changes from normal (KL grade 0) to moderate OA (KL grade 3) and minimal OA (KL grade 2) were predicted with an accuracy of 72% and 62.4%, respectively. The most predictive bone regions were identified in locations adjacent to the tibial spines.

The performance of another model, based on TBTA, for predicting the incidence of radiographic KOA was examined [8]. In this 4-year follow-up study, TBT regions selected in medial and lateral compartments were analyzed in a cohort of 203 radiographic knees, using the SDM-based roughness, degree of anisotropy, and direction of anisotropy parameters. Results from this study illustrated the ability of TBTA to predict increased medial JSN in knees with or without radiographically visible KOA at baseline. The AUC was 0.77 and 0.75 for knees with and without radiographic OA, respectively, in the medial compartment. Values of the AUC were 0.71 and 0.72 in the lateral compartment. These results were adjusted according to age, sex, and BMI [8].

In a multicentre study, the relations of TBT with JSN increase and incident radiographic KOA were examined using the VOT method [9]. Analyzing a large dataset of 1433 subjects from the Multicenter Osteoarthritis Study (MOST) [60], where most of the OA incidence and the increases in JSN occurred in the medial compartment, the results showed that a higher medial bone roughness was associated with noticeable discrimination of healthy and incident OA patients at 48- and 60-month follow-ups. The numbers of statistically significant associations found were higher for JSN increase as compared to incident OA, particularly at 30- and 60-month follow-ups. In this large dataset, TBT regions were selected using an automated method [61].

Using again the well-phenotyped population of the OAI database, it was demonstrated that TBT parameters could predict the incidence of radiographic KOA [10]. Exploiting the whole tibial proximal trabecular bone, several prediction scenarios were studied including the onset of JSN, of tibial osteophytes and the usual KL scale. Based on AUC as a global performance criterion of prediction, the inclusion of TBT parameters in the models provided AUC that ranged from 0.69 to 0.73. In addition, diagnostic odds ratios computed to examine the relevance of the models yielded high values, indicating that TBTA could identify a subset of true OA initiators with a very low number of false positives [10].

For OA incidence, all the studies reviewed here included patients with KL = 0 at baseline. However, the group of Podsiadlo also included patients with KL = 1. In terms of the TBTA method, there was no common agreement. Surprisingly, the FSA has never been employed in this regard.

The major studies related to the prediction of OA incidence are summarized in Table 3.

**Table 3:** Summary of the major studies related to the prediction of OA radiographic incidence

	Cohort name (*m*, *n*)	Period	Definition	Incidence	Major findings
Shamir et al. 2009 [35]	BLSA (Unknown, 123)	240	KL = 0	ΔKL ≥ 2	Baseline TBTA, using a Compound Hierarchy of Algorithms Representing Morphology (WND-CHARM) algorithm, of the region adjacent to the tibial spines was predictive of knee Kellgren-Lawrence (KL) incidence (best accuracy = 0.72, KL from 0 to 3)
Woloszynski et al. 2012 [8]	Lund University (CR, 105)	48	KL = 0	ΔJSN_M_ ≥ 1	Baseline TBTA, measured by the signature dissimilarity measure method (SDM), of the tibial plateau was predictive of medial knee JSN incidence (AUC = 0.75)
Podsiadlo et al. 2016 [9]	MOST (CR&RG, 1433)	48–60	KL ≤ 1	ΔJSN_M_ ≥ 1	Baseline TBTA, measured by the variance orientation transform (VOT) method, was associated with incident radiographic OA, independently of risk factors for knee OA. Most of the OA incidence occurred in medial compartments
Janvier et al. 2017 [10]	OAI (CR, 319)	48	KL = 0	ΔJSN_M_ ≥ 1	Baseline TBTA of the tibial plateau at baseline was predictive of medial knee JSN progression. The best model included TBTA, measured by the quadratic Variations estimator (Var) (AUC = 0.73)

*n* total number of included subjects, *m* image acquisition modality, *BLSA* Baltimore Longitudinal Study of Aging

#### Association of TBTA with other endpoints (prediction of knee joint replacement and responses to pharmacological treatment)

Besides KOA incidence and progression, the association of TBTA with other endpoints has also been investigated. In this regard, only two studies were detected in this review.

In the first study, published in 2014, the VOT method was employed to examine the association between baseline TBT and the risk of Knee Joint Replacement (KJR) over 6 years [45] using a sample of 114 subjects. Exclusion criteria included age of less than 40 years, WOMAC score of less than 20%, osteophyte grade of 0, KL grade of 4, or inadequate quality of the digitized radiographs for fractal analysis. If both knees were symptomatic, the knee with more severe change was excluded. The study showed that knees with a lower TBT mean FD (FD mean) at baseline had an increased risk of KJR, independent of age, gender, BMI, grade of osteophyte and JSN. This may provide a target for novel intervention strategies in this condition.

In the second study, published in 2007, the association of TBTA with treatment responses of KOA patients was evaluated [46]. 

To examine whether risedronate (an antiresorptive agent) could slow down or halt progressive JSN in OA patients, a double-blind randomized placebo-controlled study was conducted using subjects with KOA [56]. Within the framework of this study, another 2-year longitudinal radiographic study examining the effect of risedronate on subchondral bone loss in KOA patients was published, in which FSA was used to quantify longitudinal changes separately in horizontal and vertical trabeculae in ROIs selected in the medial subchondral compartment [46]. TBTA in the trial demonstrated a dose-dependent therapeutic drug effect characterized by retention of normal trabecular structure in the knee of progressors with JSN [46]. These results, whatever the clinical relevance of the use of bone–acting agents in KOA, illustrate the responsiveness of texture parameters to pharmacological treatment.

TBTA could provide markers for the enrichment of clinical trials and thereby help to enhance the screening of OA progressors and reduce the screen failure rates [15].

## Perspectives (What is unknown or should be known)

### Towards an “optimal” procedure for bone texture analysis

The above review shows that several aspects related to TBT analyses of knee radiographs have already been investigated and validated. These are summarized in this chapter and Table 4.

**Table 4:** TBT on radiographs in KOA: what is already known

*N*°	What is already known
1	TBT changes constitute one of the integral features of OA initiation and progression
2	TBT phenotype of patients with progressive OA is associated with apparent thickening of horizontal trabeculae and thinning of vertical trabeculae
3	TBT parameters evaluated from 2D radiographs reflect the subchondral trabecular bone 3D microarchitecture assessed by CT
4	Using TBT parameters in OA trials does not require supplementary methods for cross-calibration of devices at different clinical centers in addition to the standardized radiographic data acquisition procedures already performed in phase III trials

First, TBT changes constitute one of the integral features of OA initiation and progression. In this regard, different methods have been proposed and evaluated in the analysis of TBT of OA patients. These activities yielded a large number of texture descriptors without a clear group of the best performers [21], as shown in Table 5.

**Table 5:** Best TBT descriptors from selected studies, for different OA-related tasks

	Population	Major findings	Best descriptor
Messent et al. (2005) [26]	110	FSA of vertical FSA was greater in OA subjects than non-OA subjects, consistent with increased vertical trabecular number associated with thinning and fenestration of coarser trabeculae	Increase in vertical FD
Messent et al. (2005) [12]	40	No link was found between the degree of subchondral trabecular bone changes, analyzed by fractal signature analysis (FSA), and the rate of cartilage destruction, quantified by the annual rate of joint space narrowing (JSN)	Decrease in vertical FD and horizontal FD
Wolski et al. 2011 [38]	48	Significant differences in TBT, measured by that the variance orientation transform (VOT) method, between subjects with and without cartilage defects, suggesting thinning and fenestration of trabecular bone in knees with cartilage defects	Increase in vertical FD and horizontal FD
Janvier et al. (2017) [11]	1124	Baseline TBTA of the tibial plateau at baseline was predictive of medial knee JSN progression. The best model included clinical covariates, JSN and TBTA, measured by Whittle estimator (Whe) (AUC = 0.75)	Increase in vertical FD

Second, it has been demonstrated that the TBT phenotype of patients with progressive KOA is associated with apparent thickening of horizontal trabeculae and thinning of vertical trabeculae [15, 38].

Third, TBT parameters evaluated on radiographs correlate significantly with the subchondral trabecular microarchitecture assessed by μCT [41].

Fourth, when considering TBT parameters in OA trials, predictive models [11] may perform well without the need for supplementary procedures for the cross-calibration of devices at different clinical centers as standardized radiographic data acquisition procedures were usually performed in phase III trials [11, 60].

It would be of great interest to the OA research community to conduct a longitudinal study, based on a large dataset (e.g., OAI or MOST cohorts), in order to compare the four main fractal-based methods (FSA [25], Var [62], VOT [40], and Whe [63]) as well as other published TBT descriptors in order to examine the performance of these different parameters, separately or in a composite way. The prediction of OA progression or incidence has been evaluated in subsets of the two large OA cohorts (OAI and MOST) using digitized films and computed radiography. To the best of our knowledge, the effect of the modality type on the performance of prediction models has not yet been evaluated when using the different TBTA methods, with the exception of the Var method [64]. The choice of imaging modality is an important decision in routine clinical practice for diagnosis of KOA [65].

Furthermore, the selection of radiographs eligible for TBTA has not been addressed either. In the OAI cohort, several indicators are used to describe the quality of the radiographs, such as alignment, positioning, exposure problems, and visible materials and artifacts. These indicators however are not sufficient to select ROIs with the accuracy necessary for TBTA. Furthermore, in the MOST cohort, no information is included to describe the quality of radiographs (over/underexposed or with loss of details in the ROI zones). Researchers are therefore encouraged to provide sub-datasets of OAI and MOST that can be used as a gold standard for KOA progression and incidence. As examples, we suggest considering the FNIH subset proposed by Kraus et al. [15] for the prediction of OA progression in the OAI cohort, and the subset proposed by Podsiadlo et al. [9] in MOST and by Janvier et al. [10] in OAI, for the prediction of OA incidence, because of the automated procedures and the locations of bone regions.

In order to ensure the selection of radiographs on which TBTA is possible, adding a quality-control flag to OAI and MOST would be of great interest to the KOA research community.

Previous studies have reported that the tibial medial to lateral (M:L) ratio of subchondral BMD was associated with meniscal lesions and also with bone marrow lesions [66, 67]. These associations suggested that there is a link between load distribution and features of OA in knee joints. The M:L ratio of TBT descriptors was evaluated in non-radiographic OA knees after APM [37], indicating their potential for detecting early signs of OA development. However, this ratio is yet to be evaluated with radiographic OA knees.

TBT descriptors enhance the overall prediction of OA progression and incidence. However, their threshold values that provide a good trade-off between sensitivity and specificity in order to differentiate between progressors and non-progressors are still unknown.

As shown in Table 2, the prognostic capability of TBT versus changes in minimum JSW has been previously evaluated. However, researchers are encouraged to evaluate its capability versus changes in articular cartilage degeneration assessed by MRI [22], or over features of interest such as bone medullary lesions and synovitis.

There is growing interest in the use of deep learning (DL) techniques in medical imaging research [68]. DL has already shown great potential in the prediction of OA detection and progression. Recently, a DL-based method was used to estimate KL grades from plain radiographs, as part of a fully-automated model of OA detection [68, 69] and progression prediction [70]. DL-based methods have been proposed to predict the progression of radiographic joint space loss [71] and also to integrate subchondral bone texture information in the training criterion [72] to be used in the early detection of KOA. The combination of DL-based methods and TBTA was recently investigated in the OAI and MOST cohorts and showed promising results [64].

### Towards the validation of TBT as a biomarker in KOA

As reviewed above, there are several cross-sectional case–control studies that showed statistically different values of TBT parameters between KOA cases and controls [23, 24, 29, 39]. Moreover, in longitudinal studies, it was reported that TBT parameters were associated with three endpoints in KOA, namely KOA radiographic incidence [8–10], KOA progression [8, 11–15, 43, 44] or knee prosthetic surgery [45].

Taking these findings together, we now have a great body of evidence that TBT parameters are, indeed, associated and reflect alterations of the subchondral bone tissue observed in KOA. Thus, we can conclude that the biological plausibility for TBTA in KOA is already established.

Although it can be considered that TBT parameters have not been evaluated prospectively in those studies, as compared to some relevant established biological biomarkers [73], TBT parameters can be considered as imaging-based biomarkers that have been collected and acquired at the baseline and then stored and measured retrospectively in all individuals of the whole cohort, thus providing a robust assessment of the association between TBT parameters and OA endpoints studied in those cohorts (OULU [23], MOST [9], OAI [10]).

Whether TBT parameters would change consistently in response to potential OA treatment is still unclear. In [46], results obtained in terms of texture changes under the effect of risedronate could be easily compatible with the mechanism of action of an antiresorptive agent, i.e., a preservation of vertical trabecular structure in the group of patients that received risedronate 15 mg/day, and even an increase in the vertical trabecular number in the group with a higher dose (50 mg/day). Such a relationship between the use of an antiosteoporotic agent with known mechanism of action on subchondral bone tissue and changes in TBT parameters, possibly with dose-dependent effects, would be another important reason for considering TBTA as a useful biomarker for OA trials.

Finally, it is also conceivable that changes in TBT parameters under the effect of treatment could explain a relevant proportion of the preventive effect on KOA progression.

## Conclusions and research agenda

It has been reported that the association of TBT parameters with clinical (age, sex, BMI) and radiological (KL, OARSI grades) covariates improves the prediction of OA progression [11, 12, 15] and incidence [9, 10]. With the objective of providing a fully-automated prediction method, several studies focused on the automated calculation of TBT parameters and also KL [74] and JSW [75] grades. In this regard, there has been significant progress in providing computer-aided utilities for automated KOA assessment [76, 77].

The results of this review study confirm that TBTA is a relevant bioimaging marker in KOA. However, we need more knowledge to really assess what bone texture parameters are representative of, in terms of topologic or histomorphometric indices.

The research agenda would include a greater focus on the association of TBTA with the treatment responses of KOA patients. It would also include the evaluation of the association between baseline TBT and the risk of KJR using other cohorts and comparing it to the results obtained by [45]. In addition, the research agenda would involve the investigation of the performance of incidence or progression prediction models based on the TBTA of not only XR images but also their corresponding MRI images. The combination of other markers obtained from XR imaging (KL, JSW, etc.) and MRI (synovitis, bone marrow lesions, etc.) would be of interest to KOA research studies.

## Data Availability

All data generated or analyzed during this study are included in this published article.

## Abbreviations

2D: Two-dimensional
3D: Three-dimensional
ACL: Anterior cruciate ligament
APM: Arthroscopic partial meniscectomy
AUC: Area under the receiver operating characteristic curve
BLSA: Baltimore Longitudinal Study of Aging
BMD: Bone mineral density
BMI: Body mass index
BRG: Blanket with rotating grid
DL: Deep learning
DMC: Dissimilarity-based Multiple Classifier
FD: Fractal dimension
FS: Fractal signature
FSA: Fractal signature analysis
FSH: Fractal dimension calculated in the horizontal direction
FSHOT: Fractal signature Hurst orientation transform
FSV: Fractal dimension calculated in the vertical direction
HOT: Hurst orientation transform
JSN: Joint space narrowing
JSW: Joint space width
KJR: Knee joint replacement
KL: Kellgren & Lawrence
KOA: Knee osteoarthritis
LBP: Local binary pattern
M:L: Medial to lateral
MeSH: Medical Subject Headings
MOST: Multicenter Osteoarthritis Study
MRI: Magnetic resonance imaging
OA: Osteoarthritis
OARSI: Osteoarthritis Research Society International
POP: Prediction of osteoarthritis progression
PTOA: Post-traumatic osteoarthritis
ROC: Receiver operating characteristic
ROI: Region of interest
SANRA: Scale for the quality Assessment of Narrative Review Articles
SDM: Signature dissimilarity measure
Tb.Sp: Trabecular separation
TBT: Trabecular bone texture
TBTA: Trabecular bone texture analysis
Var: Quadratic variations
VOT: Variance orientation transform
Whe: Whittle
WND-CHRM: Weighted Neighbour Distance using a Compound Hierarchy of algorithms Representing Morphology
WOMAC: Western Ontario and MAcMaster
μCT: Micro–computed tomography
